# The influence of analgesic-based sedation protocols on delirium and outcomes in critically ill patients: A randomized controlled trial

**DOI:** 10.1371/journal.pone.0184310

**Published:** 2017-09-14

**Authors:** Dan Liu, Jie Lyu, Huiying Zhao, Youzhong An

**Affiliations:** Department of Critical Care, Peking University People’s Hospital, Beijing, China; UNITED STATES

## Abstract

**Objective:**

To investigate the influence of analgesic-based midazolam sedation on delirium and outcomes in critically ill patients and to analyze the risk factors of delirium.

**Design:**

Single center, prospective randomized controlled trial.

**Setting:**

A surgical intensive care unit (ICU) in a tertiary care hospital in China.

**Patients:**

Mechanically ventilated patients requiring sedation.

**Measurements and main results:**

Patients admitted to the surgical intensive care unit who required sedation and were undergoing mechanical ventilation for longer than 24 hours were randomly divided into three groups: 1) the remifentanil group received remifentanil and midazolam, 2) the fentanyl group received fentanyl and midazolam, and 3) the control group received only midazolam. The analgesic effect, sedation depth, and presence of delirium were evaluated. To compare the effect of different therapies on the occurrence of delirium, days of mechanical ventilation, length of the ICU stay, and 28-day mortality were measured along with the risk factors for delirium. A total of 105 patients were enrolled, and 35 patients were included in each group. Compared to the control group, patients who received remifentanil and fentanyl required less midazolam each day (P = 0.038 and <0.001, respectively). Remifentanil has a significant effect on reducing the occurrence of delirium (P = 0.007). The logistic regression analysis of delirium demonstrated that remifentanil (OR 0.230, 95%Cl 0.074–0.711, P = 0.011) is independent protective factors for delirium, and high APACHE II score (OR 1.103, 95%Cl 1.007–1.208, P = 0.036) is the independent risk factor for delirium.

**Conclusion:**

Remifentanil and fentanyl can reduce the amount of midazolam required, and remifentanil could further reduce the occurrence of delirium.

## Introduction

Analgesia and sedation are important therapies used in critically ill patients; however, too much sedation is associated with a longer duration of mechanical ventilation and a longer intensive care unit (ICU) stay [1]. ICU patients, particularly those with mechanical ventilation, have a rate of delirium as high as 80%, in addition to greater mortality, a longer duration of hospital stay, greater hospital costs[2,3], and poor long-term outcomes [4]. In published papers, benzodiazepine has been shown to be associated with delirium, patients from surgical[5] and burn[6] ICUs that are exposed to benzodiazepine are at increased risk of delirium (2.2 and 6.8 fold increased risk, respectively). Analgesia as a basement of sedation can reduce the amount of sedatives used, and we can thus infer that an analgesic-based sedation protocol may reduce the incidence of delirium due to a reduction in the amount of sedatives used. The purpose of this study is to investigate the influence of analgesic-based benzodiazepine sedation on delirium and outcomes in critically ill patients, in addition to the risk factors of delirium.

## Materials and methods

### Study design and participants

The study protocol was registered on www.clinicaltrials.gov (NCT02078583) and approved by the Clinical Research Ethics Committee of Peking University People’s Hospital (IRB 2013–14). Written informed consent was obtained from all patients’ legal authorized principal (the patient’s spouse, parents or children). This single center prospective randomized controlled trial was performed from September 2014 to January 2015 at Peking University People’s Hospital. The inclusion criteria were as follows: (1) signing a consent form by the patients’ legal authorized principal; (2) admission to the surgical ICU; (3) requirement for mechanical ventilation with the time of mechanical ventilation anticipated to be greater than 24 hours; (4) requirement for midazolam sedation; and (5) age greater than 18 and less than 85 years. Patients were excluded if they met any of the following criteria: (1) intracranial lesions, neurosurgical intervention, mental disabilities or coma such that they were unable to cooperate; (2) alcohol abuse; (3) history of delirium or antipsychotic use at home described according to the medical history or family members; (4) allergy to the investigational drug or other contraindications; or (5) women who were pregnant or lactating.

### Exposure

**Fig 1:**
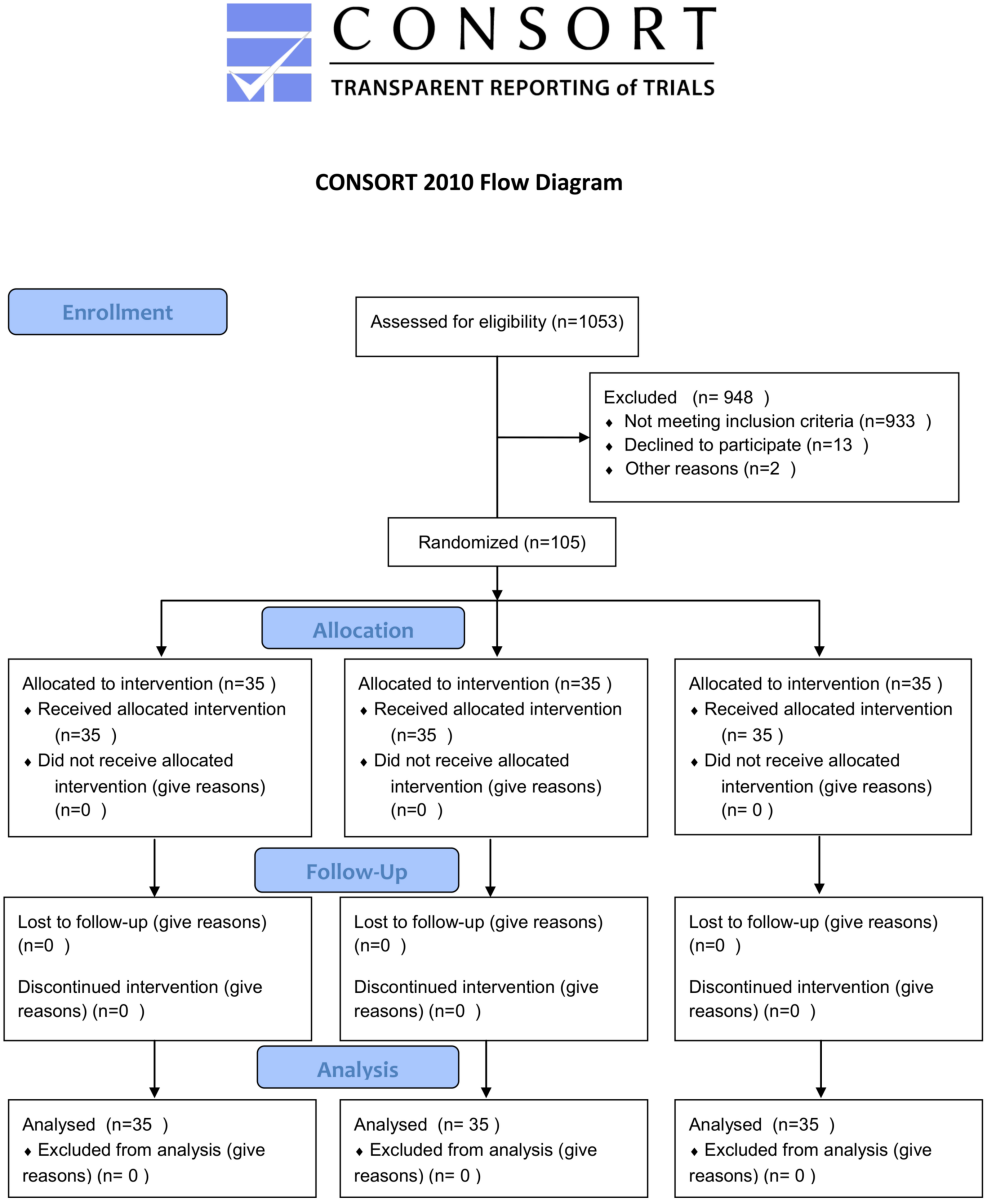
Consort 2010 flow diagram.

Fig 1 present this single center, prospective randomized controlled trial design (S1, S2 and S3 Text). A total of 105 patients were randomly allocated to three groups(1:1:1): (1) fentanyl 1μg/kg/hr and midazolam; (2) remifentanil 1μg/kg/hr and midazolam; and (3) normal saline 1μg/kg/hr and midazolam. Midazolam was administered with a loading dose of 0.05 mg/kg followed by 0.02–0.1 mg/kg/hr. The treatment administered until patients were weaned from the ventilator. Randomization was performed by the sealed envelope system, in which the study nurse randomly opened a preformed envelope containing the allocated treatment regimen. Different treatments were offered to patients in identical vials and boxes. Each box was also labeled with a numerical code, unique to treatment allocation and again blinded from both the investigator and study participant, as an additional measure to allow review of the correct treatment allocation by the study nurse. Sedation was assessed using the Richmond agitation sedation scale (RASS) every 4 hours to maintain a RASS score within -1 to -3.

### Outcomes and covariates

Baseline data including demographic characteristics, APACHE II scores within 24 hours after admission, baseline Child-Pugh classification (Table 1), and baseline creatinine were collected as well as the daily mean blood pressure (MAP) and pain scale score before and after sedation. Pain was assessed using the behavior pain scale (BPS) and the critical-care pain observational tool (CPOT). The primary endpoints are the occurrence (patient is positive for delirium at least one 8am assessment) and duration (in hours) of delirium. Sedatives were stopped to conduct daily sedation interruption every 24 hours on 8am, and then delirium was assessed by the primary nurse of each patient during this period of time using the confusion assessment method for the intensive care unit (CAM-ICU). Once the patients is diagnosed with delirium dexmedetomidine was used to release the symptom. If the patient is positive for delirium at the first 8 am assessment and treated with dexmedetomidine the primary nurse will make a every-2-hour reassessment using CAM-ICU until the patient is negative for delirium or the next day morning at 8 am. The same process is conducted every day till the patient discharge from ICU or die. The duration of delirium is the total time in hours of delirium positive. And the second endpoints are pertaining to efficacy of the analgesic (pain scales) and critical illness related outcomes: awakening time(the average duration each day from the time stopping the sedatives to the patient’s RASS score >0), weaning time(the total duration from the beginning of weaning to going off the ventilator), duration of mechanical ventilation (the total hours when the patient is ventilated), length of ICU stay, and 28-day mortality(all patients are followed-up till die or the 28^th^ day from the adimission to the ICU).

**Table 1:** Child-Pugh score.

Indicator	1 point	2 points	3 points
Total Serum Bilirubin	<2 mg/dl	2–3 mg/dl	>3 mg/dl
Serum Albumin	>3.5 g/dl	2.8 to 3.5 g/dl	<2.8 g/dl
INR	<1.70	1.71 to 2.20	>2.20
Ascites	No Ascites	Ascites controlled medically	Ascites poorly controlled
Encephalopathy	No Encephalopathy	Encephalopathy controlled medically	Encephalopathy poorly controlled

Child Class A (5 to 6 points): Life expectancy is 15 to 20 years and abdominal surgery peri-operative mortality is 10%; Child Class B (7 to 9 points): Indicated for liver transplantation evaluation and abdominal surgery peri-operative mortality: 30%; Child Class C(10 to 15 points): Life expectancy is 1 to 3 years and abdominal surgery peri-operative mortality is 82%

### Statistical analysis

A sample size of 90 patients was expected to provide 90% power (two-sided, α = 0.05, β = 0.10) for detection of a significant difference about absolute 35% decrease of delirium rate in remifentanil group compared to the control group with hypothesized delirium rate of 46%. To anticipate potential drop-out rate of 15%, we aimed to include a total of 105 patients, 35 patients in each group. Continuous variables are presented as the means ± standard deviation (SD) or as medians (inter-quartile ranges). The differences between multiple samples were compared using one-way analysis of variance (ANOVA), and the differences between two samples were compared using an independent samples t-test or a Mann-Whitney U test. Categorical variables are presented as the number of patients (percentage), and data were compared using a chi-square test or Fisher’s exact test. According to whether the patient is delirious or not, dead or survival a subgroup analyses is conduct. A multivariate logistic regression model was used to determine the risk factors for delirium. Lowess plots of delirium as a function of the continuous exposures was made before the logistic regression, then Hosmer Lemeshow test and observed vs. predicted graph of delirium are made to judge the fit of the model. Two-sided P values less than 0.05 were regarded as significant. Statistical analyses were performed using the SPSS 16.0 software for windows (SPSS Inc., Chicago, IL, USA).

## Results

### Demographic of the patients

105 patients were included in this study, with 35 patients in each group, all the patients included received the intended treatment and the outcomes were analyzed. No differences were found in the patient characteristics among the three groups with regard to sex, age, body weight, surgical type, severity of illness (APACHE II score), baseline liver and renal function and the situation of septic shock (Table 2).

**Table 2:** Patient characteristics.

Characteristics	Remifentanil (n = 35)	Fentanyl (n = 35)	Control (n = 35)	P values
Age (years)	66.11±11.94	62.00±9.96	64.49±10.01	0.272
Sex (male/female)	21/14	17/18	17/18	0.543
Body weight (kg)	65.29±17.54	67.66±9.95	65.69±12.33	0.739
Disease (n (%))				
Abdominal	21 (60.0)	17 (48.6)	15 (42.9)	0.331
Vascular	8 (22.9)	10 (28.6)	8 (22.9)	
Orthopedic	3 (8.6)	3 (8.6)	1 (2.9)	
Genitourinary	2 (5.7)	5 (14.3)	9 (25.7)	
Others	1 (2.9)	0 (0)	2 (5.7)	
APACHE II score	19.20±4.19	20.20±5.04	21.11±6.62	0.334
Child-Pugh classification (A/B/C)	26/8/1	24/8/3	24/7/4	0.746
Creatinine (μmol/l)	70 (50, 92)	75 (55, 112)	68 (55, 83)	0.963
Septic shock (n (%))				
Yes	24 (68.6)	28 (80.0)	25 (71.4)	0.532
No	11 (31.4)	7 (20.0)	10 (28.6)	

### Occurrence and duration of delirium

Significant differences were noted in the delirium rate among the three groups (P = 0.014, 22.9% for the remifentanil group, 40% for the fentanyl group, and 57.1% for the control group). Compared to the control group, patients in the remifentanil group had a significantly lower rate of delirium (P = 0.007). No other statistical intergroup differences were found. Although the rate of delirium in the fentanyl group was less than that in the control group, it was not statistically significant; this was also the case when the remifentanil group was compared to the fentanyl group. Therefore, we can infer that compared to fentanyl, remifentanil has an advantage in reducing delirium. We did not observe any significant differences in the duration of delirium among the three groups (P = 0.494); however, patients who received remifentanil tended to have the shortest duration of delirium. (Table 3).

**Table 3:** Outcomes.

Characteristics	Remifentanil (n = 35)	Fentanyl (n = 35)	Control (n = 35)	P values
Delirium (n (%))	8 (22.9)	14 (40.0)	20 (57.1)	0.014
Duration of delirium (hours)	147.00 (121.25,169.00)	180.50 (104.75,339.00)	165.50 (100.75,260.50)	0.494
MAP_before_(mmHg)	82 (73, 95)	81 (71, 93)	85 (67, 97)	0.485
MAP_after_ (mmHg)	81 (75, 88)	78 (71, 87)	80 (71, 91)	0.748
MAP_after-before_(mmHg)	1(-11,8)	-3(-15,9)	-2(-11,6)	0.963
BPS_before_	4 (3, 4)	4 (4, 5)	4 (4, 5)	0.143
BPS_after_	3 (3, 3)	3 (3, 3)	3 (3, 3)	0.076
BPS_after-before_	-1(-1,0)	-1(-2,-1)	-1(-2,-1)	0.090
CPOT_before_	3 (2, 4)	4 (3, 4)	4 (3, 4)	0.497
CPOT_after_	0 (0, 0)	0 (0, 0)	0 (0, 0)	0.055
CPOT_after-before_	-3(-4,-2)	-4(-4,-2)	-3(-4,-2)	0.560
Daily midazolam (mg)	160.57±32.95	146.76±31.86	178.95±43.73	0.002
Daily analgesics (mg)	98.59±24.98	96.4±14.09		0.653
Daily dexmedetomidine (mg)	0.43±0.12	0.65±0.22	0.62±0.14	0.036
Awakening time (hours)	2.360±0.92	2.51±1.06	2.43±0.82	0.783
Weaning time (hours)	12 (6.25,29.50)	18 (8.00,59.00)	10 (6.00,35.00)	0.670
Duration of MV (hours)	102 (68,157)	126 (68,256)	114 (65,188)	0.485
Length of ICU stay (days)	6 (4,9)	7 (5,13)	7 (5,13)	0.540
28-day all-cause mortality (n (%))	4 (11.4%)	4 (11.4%)	7 (20.0%)	0.497

MAP_before_: mean artery preasure before sedation. MAP_after_: mean artery preasure after sedation. MAP_after-before_: mean artery preasure changes after compared to before sedation. BPS_before_: behavior pain scale before sedation. BPS_after_: behavior pain scale after sedation. BPS_after-before_: behavior pain scale changes after compared to before sedation. CPOT_before_: the critical-care pain observational tool before sedation. CPOT_after_: the critical-care pain observational tool after sedation. CPOT_after-before_: the critical-care pain observational tool changes after compared to before sedation. MV: mechanical ventilation. ICU: intensive care unit

### Efficacy of analgesia

Significant differences were observed in the amount of daily sedatives that the patients received in the three groups (P = 0.002). Compared to the control group (178.95±43.73 mg/day,), the patients in remifentanil (160.57±32.95 mg/day, P = 0.038) and fentanyl (146.76±31.86 mg/day, P = <0.001) groups required less midazolam each day. The difference between remifentanil and fentanyl groups was not statistically significant. Patients receiving fentanyl had the lowest daily midazolam consumption. No difference was found in the amount of analgesics administered between fentanyl and remifentanil groups. Patients who developed delirium were treated with dexmedetomidine, and a significant difference was found for the daily amount of dexmedetomidine administered within the three groups (P = 0.036). Compared to the fentanyl group (0.65±0.22 mg/day, P = 0.05) and the control group (0.62±0.14 mg/day, P = 0.007), the remifentanil group had the lowest daily dexmedetomidine intake (0.43±0.12 mg/day), and no difference was found between the fentanyl and control groups (P = 0.782). No significant differences in pain score were observed before and after sedation among the three groups, and no difference was found in the mean blood pressure. From these results, we can infer that the efficacy and safety of analgesia is the same in the three groups. (Table 3).

### Characteristics of critical illness related to outcomes

No significant differences were found in the awakening time (P = 0.783), weaning time (P = 0.670) and duration of mechanical ventilation (P = 0.485) among the three groups; however, compared to the control group, a trend was noted that patients who received remifentanil had a shorter duration of awakening time, weaning time, and mechanical ventilation. Conversely, fentanyl showed a trend toward increasing the awakening and weaning time along with the duration of mechanical ventilation. No significant differences were observed for the length of ICU stay among the groups (0.540). Regarding 28-day mortality, a trend was noted in which fentanyl- or remifentanil-based sedation produced a lower rate than observed in the control group, but this trend was not statistically significant (P = 0.497) (Table 3).

### Risk factors for delirium

42 (40%) of the 105 patients developed delirium. Compared to those without delirium, no differences were found regarding age, sex, body weight, surgical type, basal renal function and the condition of septic shock. Patients with delirium had greater APACHE II scores (22.23±5.65 vs. 18.79±4.76. P = 0.001), and this group included more patients with a Child-Pugh classification of B or C (42% vs. 20.6%, P = 0.013). Delirious patients had a lower rate of analgesic-based sedation therapy (52.4% vs.76.2%, P = 0.019). Delirium was associated with a prolonged awakening time (2.65±0.99 vs. 2.29±0.87 hours, P = 0.047), weaning time [25.50 (8.38,50.75) vs. 10 (6.38,28.75) hours, P = 0.049],duration of mechanical ventilation [157.00 (100.75,353.50) vs. 86.00 (59.00,143.00) hours, P = 0.001], length of ICU stay [8.50 (5.00,24.25) vs. 6.00 (5.00,8.00) days, P = 0.005]and an increased 28-day mortality rate (33.3%vs.1.6%, P<0.001) (Table 4). Lowess plots of delirium as a function of the continuous exposures was made before the logistic regression analysis of delirium. For APACHE II score see S1 Fig and creatinine see S2 Fig. A plot of observed vs. predicted graph shows the model of good fit (S3 Fig). Multiple logistic regression analysis (Hosmer and Lemeshow Test of the logistic regression model is: Chi-square is 10.327, P = 0.24) identified remifentanil combined with midazolam therapy (OR 0.230, 95%Cl 0.074–0.711, P = 0.011) as an independent protective factor for delirium, high APACHE II score (OR 1.103, 95%Cl 1.007–1.208, P = 0.036) is the independent risk factor for delirium. (Table 5).

**Table 4:** Delirious vs. non-delirious patients.

Characteristics	Delirious (n = 42)	Non delirious (n = 63)	P value
Age (years)	65.52±11.93	63.32±9.82	0.323
Sex (male/female)	25/17	30/33	0.319
Body weight (kg)	67.67±11.95	65.24±14.53	0.371
Disease (n (%))			0.293
Abdominal	23 (54.8)	30 (47.6)	
Vascular	12 (28.6)	14 (22.2)	
Orthopedic	2 (4.8)	5 (7.9)	
Genitourinary	3 (7.1)	13 (20.6)	
Others	2 (4.8)	1 (1.6)	
APACHEIIscore	22.23±5.65	18.79±4.76	0.001
Child-Pugh classification (A/B/C)	24/12/6	59/11/2	0.026
Creatinine (μmol/l)	69.00 (55.00,104.00)	71.00 (48.50,104.25)	0.143
Septic shock (n (%))			
Yes	34 (81.0)	43 (68.3)	0.180
No	8 (19.0)	20 (31.7)	
Analgesic-based sedation n (%)	22 (52.4)	48 (76.2)	0.019
Daily midazolam (mg)	166.17±42.54	159.38±35.77	0.379
Daily analgesics (mg)	100.86±14.67	95.95±22.26	0.348
Awakening time (hours)	2.65±0.99	2.29±0.87	0.047
Weaning time (hours)	25.50 (8.38,50.75)	10 (6.38,28.75)	0.049
Duration of MV (hours)	157.00 (100.75,353.50)	86.00 (59.00,143.00)	0.001
Length of ICU stay (days)	8.50 (5.00,24.25)	6.00 (5.00,8.00)	0.005
28-day all-cause mortality n (%)	14 (33.3)	1 (1.6)	<0.001

MV: mechanical ventilation. ICU: intensive care unit

**Table 5:** Risk factors for delirium.

	OR	95% CI		P
APACHE II score	1.103	1.007	1.208	0.036
Child-Pugh score				
Child-Pugh (A)	1.494	0.495	4.509	0.476
Child-Pugh (B)	3.485	0.564	21.556	0.179
Creatinine (μmol/l)	1.006	0.997	1.015	0.218
Septic shock	0.641	0.224	1.832	0.406
group				
Remifentanil+midazolam	0.230	0.074	0.711	0.011
Fentanyl +midazolam	0.431	0.150	1.241	0.119

### Deceased vs. surviving patients

15 (14.29%) of the 105 patients died, and no differences were observed in those patients compared to the surviving patients with respect to age, sex, body weight, surgical type, baseline hepatic and renal function as well as the condition of septic shock. The deceased patients had greater APACHE II scores (19.67±5.11 vs. 23.20±6.12, p = 0.018), a lower percentage of analgesic-based sedation therapy (40% vs. 68.9%, P = 0.041). The rate of delirium was significantly greater in the deceased patients (93% vs. 31.3%, P<0.001). No differences were found in the duration of delirium, awakening time, weaning time, and mechanical ventilation and the length of ICU stay (Table 6).

**Table 6:** Deceased vs. surviving patients.

Characteristics	Surviving (n = 90)	Deceased (n = 15)	P value
Age (years)	62.24±10.87	63.93±10.05	0.918
Sex (male/female)	48/42	7/8	0.782
Body weight (kg)	66.69±13.92	63.33±11.09	0.377
Disease (n (%))			
Abdominal	45(50.0)	8(53.3)	0.051
Vascular	22(24.4)	4(26.7)	
Orthopedic	6(6.7)	1(6.7)	
Genitourinary	16(17.8)	0(0.0)	
Others	1(1.1)	2(13.3)	
APACHEIIscore	19.67±5.11	23.20±6.12	0.018
Child-Pugh classification (A/B/C)	64/21/5	10/2/3	0.126
Creatinine (μmol/l)	70.50 (55.00,105.25)	61.00 (47.00,82.00)	0.250
Septic shock (n (%))			
Yes	64(71.1)	13(86.7)	0.344
No	26(28.9)	2(13.3)	
Analgesic based sedation n(%)	62 (68.9)	6 (40.0)	0.041
Daily midazolam (mg)	162.99±36.68	156.73±49.59	0.563
Daily analgesics (mg)	97.95±20.74 (n = 62)	93.96±15.58 (n = 8)	0.602
Delirium n (%)	28 (31.1)	14 (93.3)	<0.001
Duration of delirium (hours)	168.00 (111.25,282.75)	166.00 (102.25,243.25)	0.823
Awakening time (hours)	2.50 (1.50,3.00)	2.50 (1.50,3.50)	0.234
Weaning time (hours)	12.50 (7.00,35.25)	7.00 (2.00,12.00)	0.276
Duration of MV (hours)	105.50 (65.75,161.75)	184.00 (103.00,352.00)	0.076
Length of ICU stay (days)	7.00 (5.00,11.25)	8.00 (4.00,15.00)	0.993

MV: mechanical ventilation. ICU: intensive care unit

## Discussion

Both acute and chronic mental dysfunction, especially those related to analgesics and sedatives have attracted increasing attention. Delirium is a syndrome characterized by disturbances of consciousness, attention, cognition, and perception that develops over a short period and tends to fluctuate throughout the day. It is the most common form of acute mental dysfunction in critically ill patients and has been defined as the sixth vital sign that should be routinely assessed routinely [7]. The PAD guidelines (Clinical Practice Guidelines for the Management of Pain, Agitation, and Delirium in Adult Patients in the Intensive Care Unit) in 2013 made delirium an important consideration and recommended routine monitoring of delirium in adult ICU patients [8]. Data regarding the relationship between benzodiazepine and delirium is consistent. Studies from medical [9], surgical trauma [10] and burn [6] ICUs have revealed that benzodiazepine use may be a risk factor for the development of delirium in adult ICU patients. In our study, we found the patients who received midazolam sedation without analgesics had a rate of delirium as great as 57.1%. The PAD guidelines suggest that sedation strategies using nonbenzodiazepine sedatives (either propofol or dexmedetomidine) may be preferred over sedation with benzodiazepines (either midazolam or lorazepam) to improve clinical outcomes in mechanically ventilated adult ICU patients [8]. The data are insufficient to determine the relationship between propofol use and the development of delirium in adult ICU patients [9]. Furthermore a prolonged infusion of propofol leads to hyperlipidemia and for patients with unstable hemodynamic are more susceptible to hypotension. Some trials have demonstrated that dexmedetomidine maybe associated with a lower prevalence of delirium than benzodiazepine infusions [11, 12]. However, for patients who require deep sedation, dexmedetomidine will not achieve the desired sedative depth. Furthermore, both propofol and dexmedetomidine cost much more than benzodiazepines. Therefore, the use of benzodiazepines, especially in patients with unstable hemodynamic requiring deep sedation, is inevitable. We performed this study to determine how to reduce the incidence of delirium associated with benzodiazepines and, specifically, to investigate whether analgesic-based sedation protocols can reduce delirium in ventilated critically ill patients with midazolam sedation.

Insufficient analgesia results in worsening stress, sleep deprivation, cognitive dysfunction, anxiety, even delirium and post-traumatic stress disorder (PTSD) [13–15]. The synergistic effect of analgesia and sedation is reflected by the fact that analgesics can reduce the amount of sedatives required [16]. In this study, we observed that fentanyl and remifentanil cause a significant reduction in the required dose of midazolam.

More recently, some studies have focused on analgesic-based sedation protocols. Rozendaal [17]found that remifentanil together with propofol, given when necessary, compared to propofol or midazolam together with opiates when necessary results in shorter ICU length of stay and duration of ventilation and better sedation-agitation scores (SAS). Other studies that compared analgesic-based sedation to traditional sedation revealed a significant reduction in the duration of mechanical ventilation [18, 19]. A single center randomized control trial compared no sedation (opiates only for analgesia) with sedation (20 mg/ml propofol for 48 h, 1 mg/mL midazolam thereafter), and patients receiving no sedation had significantly more days without ventilation. No difference was observed in the occurrence of accidental extubation or ventilator-associated pneumonia [20]. Therefore, analgesia is of great importance. Conflicting data exist with respect to the relationships between delirium and opiates. One study inferred that fentanyl is a risk factor for delirium in surgical and traumatic ICU patients [10]. However, some researchers have found that fentanyl can reduce the occurrence of delirium [6]. Remifentanil and delirium have rarely been investigated. The results from patients in the post anesthesia care unit (PACU) have shown that, compared to fentanyl, remifentanil reduced delirium in post-operative patients [21]. Another study evaluated the effect of different analgesics (fentanyl, sufentanil, and remifentanil) combined with dexmedetomidine on mechanically ventilated patients, remifentanil combined with dexmedetomidine reduced the occurrence of delirium [22]. In our study, we found that compared to sedation using midazolam only, remifentanil, when it is used as an analgesic, combined with midazolam sedation can significantly reduce the rate of delirium, whereas fentanyl showed an insignificant trend. From these results, we can infer that remifentanil has an advantage over fentanyl with respect to preventing the development of delirium. Furthermore, remifentanil can reduce the use of dexmedetomidine prescribed to address delirium. That is patients who receive remifentanil once delirium developed, the delirium would be simple to treat. Remifentanil is a potent μ-receptor agonist with the unique features of rapid onset and rapid predictable offset of action, which makes it quickly adjustable to the required level of analgesia. A randomized control trial revealed that remifentanil is superior with respect to awakening, reducing sedatives, and extubation time compared to morphine [16]. We found that remifentanil had the equivalent analgesic effect of fentanyl, which was manifested by no differences in the pain scale before and after treatment, and no differences were found regarding side effects, such as hypotension. We can infer that midazolam sedation based on adequate analgesia can reduce delirium and has good safety. The results of our study showed that fentanyl has a potential effect of prolonging the awakening time and duration of mechanical ventilation; therefore, remifentanil may have an advantage over fentanyl in mechanically ventilated patients. Analgesic-based sedation did not significantly improve the ICU length of stay compared to the control group. We observed a trend for remifentanil and fentanyl to improve the 28-day mortality, however it was not statistically significant.

In our study, we observed that delirious patients had higher APACHE II scores, which is consistent with a previous study [23], and a poorer Child-Pugh classification, from which we could identify poor liver function as a predictor of delirium. The logistic regression demonstrated that remifentanil combined with midazolam is the independent protective factor for delirium. From this finding, we could infer that administering opioids, especially remifentanil, as a basic analgesic treatment could significantly reduce the occurrence of delirium in patients receiving midazolam. We found that the deceased patients had higher APACHE II scores which implies that the severity of the illness may increase the risk of death. We also found that deceased patients had lower percentage of analgesic-based sedation therapy, therefore, we could infer that, for patients receiving midazolam, administering opioids analgesia could effectively reduce the occurrence of delirium and may further improve the mortality rate at 28 days.

The shape of the Lowess plots are not smooth enough may due to the small sample size and relative few patients with delirium. However both the Hosmer Lemeshow test and observed vs. predicted graph demonstrate the logistic regression model of good fit.

Some limitations of our study exist. First, all patients received midazolam; therefore, the results cannot be applied to other sedatives, such as propofol or dexmedetomidine. Second, patents in our study were all surgical ICU patients; the findings cannot be applied to other ICU patients. In addition, this is a single center trial with a small sample; the results deserve further confirmation in trials at multiple centers with a large sample.

## Conclusion

Patients who received benzodiazepines have a relatively greater risk of delirium; analgesics can reduce the amount of sedatives required and can further reduce the occurrence of delirium and improve the prognosis. Remifentanil may have an advantage over fentanyl in reducing delirium.

## Supporting information

S1 Text CONSORT 2010 checklist. (DOC)

S2 Text Plosone protocol English. (DOCX)

S3 Text Plosone protocol Chinese. (DOC)

S1 Data set Primary data. (XLSX)

S1 Fig Lowess Plots APACHE II score. (PNG)

S2 Fig Lowess Plots creatinine. (PNG)

S3 Fig Plots of ob vs. pre graph. (PNG)
