# Supplementary material for: The influence of analgesic-based sedation protocols on delirium and outcomes in critically ill patients: A randomized controlled trial
Source: PLoS One. 2017 Sep 14;12(9):e0184310. doi: 10.1371/journal.pone.0184310 (PMC5598969; doi:10.1371/journal.pone.0184310)
Supplement: S2 Text — (DOCX) [file pone.0184310.s002.docx]

**PROTOCAL （ENGLISH）**

**(Version submitted to and approved by ethics committee before start of trial)**

**Title：The influence of analgesic-based sedation protocols on delirium and outcomes in critically ill patients**

**Study type：Single center, prospective randomized controlled trial**

**Investigators：Youzhong An（MB）**

**Dan Liu（MD）**

**JIe Lyu （MD）**

**Huiying Zhao （MD）**

**Introduction**

Analgesia and sedation are of vital importance for critically ill patients, and when given appropriately, can relieve pain, anxiety and agitation, reduce oxygen consumption, and improve the balance between oxygen supply and consumption. However, too much sedation can lead to prolonged mechanical ventilation and a longer ICU stay (1).

Both acute and chronic mental dysfunction, especially those related to analgesics and sedatives, have attracted increasing attention. Delirium is a syndrome characterized by disturbances of consciousness, attention, cognition, and perception that develops over a short period and tends to fluctuate throughout the day. It is the most common form of acute mental dysfunction in critically ill patients and has been defined as the sixth vital sign that should be assessed routinely (2). ICU patients are more susceptible to delirium; it has been reported that delirium affects up to 80% patients that are mechanically ventilated following a long-term cognitive disorder (3).Delirium is associated with higher mortality, a longer hospital stay, and greater hospital cost (4,5).Therefore, measures are needed to achieve better prevention and treatment. Data regarding the relationship between benzodiazepine and delirium is consistent. Studies from medical (6), surgical trauma (7) and burn (8) ICUs have revealed that benzodiazepine use may be a risk factor for the development of delirium in adult ICU patients.

Insufficient analgesia results in worsening stress, sleep deprivation, cognitive dysfunction, anxiety, and even delirium and post-traumatic stress disorder (PTSD) (9-11). Remifentanil is a potent μ-receptor agonist with the unique features of rapid onset and rapid predictable offset of action, which makes it quickly adjustable to the required level of sedation. A randomized control trial revealed that remifentanil is superior with respect to awakening, reducing sedatives, and extubation time compared to morphine (12). Conflicting data exist with respect to the relationships between delirium and opiates. One study inferred that fentanyl is a risk factor for delirium in surgical and traumatic ICU patients (7).However, some researchers have found that fentanyl can reduce the occurrence of delirium (8). Remifentanil and delirium have rarely been investigated.

More recently, some studies have focused on analgesic-based sedation protocols. Rozendaal (13) found that remifentanil together with propofol, given when necessary, compared to propofol or midazolam together with opiates when necessary results in shorter ICU length of stay and duration of ventilation and better sedation-agitation scores (SAS). Other studies that compared analgesic-based sedation with traditional sedation revealed a significant reduction in the duration of mechanical ventilation (14,15). A single center randomized control trial compared no sedation (opiates only for analgesia) with sedation (20 mg/mL propofol for 48 h, 1 mg/mL midazolam thereafter), and patients receiving no sedation had significantly more days without ventilation. No difference was observed in the occurrence of accidental extubations or ventilator-associated pneumonia (16).Therefore, analgesia is of great importance.

As mentioned above, benzodiazepine is a potential risk factors for delirium ,and the synergistic effect of analgesia and sedation is reflected by the fact that analgesics can reduce the amount of sedatives required, so may also reduced the prevalence of delirium associated with the use of benzodiazepines. Based on this consumption, we designed this trail to evaluate the influence of analgesic based benzodiazepine sedation on delirium in critically ill patients.

**Aims:** To investigate the influence of analgesic-based midazolam sedation on delirium and outcomes in critically ill patients

**Material and methods:**

***Study design***

Single center, prospective randomized controlled trial**.**

***Study population***

A sample size of 88 patients was expected to provide 90% power (two-sided, α =0.05) for detection of a significant difference of delirium rate within different groups. To anticipate potential drop-out, we aimed to include a total of 105 patients, 35 patients in each group.

***Inclusion criteria:***

(1) Signing a consent form;

(2) Admission to the surgical ICU;

(3) Requirement for mechanical ventilation with the time of mechanical ventilation anticipated to be greater than 24 hours;

(4) Requirement for midazolam sedation;

(5) Age greater than 18 and less than 85 years.

***Exclusion criteria:***

(1) Intracranial lesions, neurosurgical intervention, and mental disabilities such that they were unable to cooperate;

(2) Alcohol abuse;

(3) History of delirium or antipsychotic use at home described according to the medical history or family members;

(4)Allergy to the investigational drug or other contraindications;

(5) Women who were pregnant or lactating.

***Intervention***

The patients were randomly allocated to three groups: (1) fentanyl 1μg/kg/hr and midazolam; (2)remifentanil 1μg/kg/hr and midazolam; and(3) normal saline 1μg/kg/hr and midazolam. Midazolam was administered with a loading dose of 0.05 mg/kg followed by 0.02-0.1 mg/kg/hr. Pain was assessed using the behavior pain scale (BPS) and the critical-care pain observational tool (CPOT), and sedation was assessed using the Richmond agitation sedation scale (RASS) every 4 hours to maintain a RASS score within -1 to -3. Sedatives were stopped to conduct daily sedation interruption every 24 hours on 8am ,and then delirium was assessed during this period of time using the confusion assessment method for the intensive care unit (CAM-ICU).Once the patients is diagnosed with delirium dexmedetomidine was used to release the symptom.

***Data collection***

Baseline data including demographic characteristics, APACHE II scores within 24 hours after admission, baseline Child-Pugh classification, and baseline creatinine were collected as well as the mean blood pressure and pain scale score before and after sedation. Primary endpoints are the occurrence (defined by the percentage of patients with delirium in each group) and duration(between the first appearance of delirium symptom to its disappear ) of delirium , and the second endpoints are awakening time(duration from the time stopping the sedatives to the patient’s RASS score ＞0), weaning time, duration of mechanical ventilation, length of ICU stay, and 28-day mortality.

**Reference**

. Kollef MH, Levy NT, Ahrens TS, Schaiff R, Prentice D, Sherman G. The use of continuous i.v. sedation is associated with prolongation of mechanical ventilation. Chest. 1998;114(2):541–548.

2. Flaherty JH, Rudolph J, Shay K, et al. Delirium is a serious and under-recognized problem: why assessment of mental status should be the sixth vital sign. J Am Geriatr Soc 2007;8(5):273–5.

3. Morandi A, Jackson JC. Delirium in the intensive care unit: a review. Neurol Clin. 2011;29(4):749–763.

4. Pisani MA, Kong SY, Kasl SV, Murphy TE, Araujo KL, Van Ness PH. Days of delirium are associated with 1-year mortality in an older intensive care unit population. Am J Respir Crit Care Med. 2009; 180(11):1092–1097.

5. Shehabi Y, Riker RR, Bokesch PM, Wisemandle W, Shintani A, Ely EW. Delirium duration and mortality in lightly sedated, mechanically ventilated intensive care patients. Crit Care Med. 2010;38(12):2311–2318.

6.Pandharipande P, Shintani A, Peterson J, etal. Lorazepam is an independent risk factor for transitioning to delirium in intensive care unit patients. Anesthesiology. 2006;104(1):21–26.

7. Pandharipande P, Cotton BA, Shintani A, etal. Prevalence and risk factors for development of delirium in surgical and trauma intensive care unit patients. J Trauma. 2008;65(1):34–41.

8.Agarwal V, O’Neill PJ, Cotton BA, etal. Prevalence and risk factors for development of delirium in burn intensive care unit patients. J Burn Care Res. 2010;31(5):706–715.

9. Kapfhammer HP, Rothenhausler HB, Krauseneck T, Stoll C, Schelling G. Posttraumatic stress disorder and health-related quality of life in long-term survivors of acute respiratory distress syndrome. Am J Psychiatry. 2004;161(1):45–52.

10. Morrison RS, Magaziner J, Gilbert M, etal. Relationship between pain and opioid analgesics on the development of delirium following hip fracture. J Gerontol A Biol Sci Med Sci. 2003;58(1):76–81.

11. Rotondi AJ, Chelluri L, Sirio C, etal. Patients’ recollections of stressful experiences while receiving prolonged mechanical ventilation in an intensive care unit. Crit Care Med. 2002;30(4):746–752.

12. Dahaba AA, Grabner T, Rehak PH, List WF, Metzler H. Remifentanil versus morphine analgesia and sedation for mechanically ventilated critically ill patients: a randomized double blind study. Anesthesiology. 2004;101(3):640–646.

3. Rozendaal FW, Spronk PE, Snellen FF, etal. Remifentanil-propofol analgo-sedation shortens duration of ventilation and length of ICU stay compared to a conventional regimen: a centre randomised, cross-over, open-label study in the Netherlands. Intensive Care Med. 2009;35(2): 291–298.

14. Breen D, Karabinis A, Malbrain M, etal. Decreased duration of mechanical ventilation when comparing analgesia-based sedation using remifentanil with standard hypnotic-based sedation for up to 10days in intensive care unit patients: a randomised trial [ISRCTN47583497]. Crit Care. 2005;9(3):R200–R210.

15. Karabinis A, Mandragos K, Stergiopoulos S, etal. Safety and efficacy of analgesia-based sedation with remifentanil versus standard hypnotic-based regimens in intensive care unit patients with brain injuries: a randomized, controlled trial [ISRCTN50308308]. Crit Care. 2004; 8(4):R268–R280.

16. Strom T, Martinussen T, Toft P. A protocol of no sedation for critically ill patients receiving mechanical ventilation: a randomised trial. Lancet. 2010;375(9713):475–480.
