# Supplementary material for: The influence of analgesic-based sedation protocols on delirium and outcomes in critically ill patients: A randomized controlled trial
Source: PLoS One. 2017 Sep 14;12(9):e0184310. doi: 10.1371/journal.pone.0184310 (PMC5598969; doi:10.1371/journal.pone.0184310)
Supplement: S3 Text — (DOC) [file pone.0184310.s003.doc]

**以镇痛为基础的镇静治疗对危重症患者谵妄影响的研究**

**一、背景**

镇痛和镇静治疗特指应用药物手段以消除患者疼痛，减轻患者焦虑和躁动，催眠并诱导顺行性遗忘的治疗，降低危重症患者氧耗，改善氧供和氧耗平衡，在重症加强治疗病房(ICU)患者基本治疗中占有举足轻重的地位，目前已得到广泛的认可并成为危重症患者一种常规的治疗手段。但过度镇静在ICU中十分常见，并造成机械通气时间和ICU住院天数延长【[[1]](#endnote-2)】。

危重症患者治疗的最终目标在于靶器官保护，其中危重症患者的急慢性脑功能障碍已成为一种复杂且严峻的社会问题摆在我们面前，而近年来镇痛镇静药物所引起的脑功能障碍也越来越引起大家的重视。谵妄作为急性脑功能障碍最为常见的表现形式之一成为研究者关注的焦点，并且已有学者提出将对于患者神经系统功能特别是谵妄状态的评估最为“第六生命体征”进行常规监测【[[2]](#endnote-3)】。ICU内谵妄的发生率高达80%，并将引起患者远期的认知功能障碍【[[3]](#endnote-4)】。谵妄导致患者死亡率及病死率增加，住院天数延长，住院花费增加【[[4]](#endnote-5)】【[[5]](#endnote-6)】。可见对于谵妄引起足够的重视并采取必要的预防和治疗措施已迫在眉睫。镇痛镇静治疗与谵妄的发生之间存在一定的关系。其中，镇静治疗与谵妄之间的关系比较一致，目前来自内科【[[6]](#endnote-7)】、外科、创伤【[[7]](#endnote-8)】及烧伤【[[8]](#endnote-9)】ICU的多项研究结果显示，应用最为广泛的苯二氮卓类镇静药物与谵妄之间存在明确的相关性。

镇痛镇静二者密不可分，以镇痛为基础的镇静治疗这一概念早在多年前就已提出。危重症患者存在包括机械通气、有创监测、原发疾病、日常护理及医疗操作等多种多导致疼痛不适的原因，镇痛不足将加重应激反应、引起睡眠不足、定向力异常、焦虑甚至包括谵妄和创伤后应激（PTSD）在内的脑功能障碍【[[9]](#endnote-10)】【[[10]](#endnote-11)】【[[11]](#endnote-12)】。但由于考虑到药物副作用（呼吸系统及血流动力学抑制）及阿片类药物成瘾等因素，以及缺乏理想的疼痛评估手段，导致疼痛治疗并没有得到应有的重视。近年来随着镇静技术的发展以及新型阿片类药物的研制，使得镇痛治疗再次受到关注。瑞芬太尼是新型阿片类药物的代表之一，这是一种纯粹的μ受体激动剂，被血液或组织中的非特异性酯酶水解代谢，不依赖肝肾代谢清除，起效快，迅速达峰效应，持续时间短，即使长时间应用无蓄积。与传统的阿片类药物（芬太尼、吗啡等）相比有一定的先进性。但在ICU患者镇痛药物的选择方面鲜有研究。一项随机双盲研究结果显示，与吗啡相比瑞芬太尼在达到最佳觉醒时间、减少镇静药物使用、拔管时间方面显示出一定的优势【[[12]](#endnote-13)】。目前的资料显示，阿片类镇痛药物与谵妄之间的关系并不确定且证据尚不确凿。有资料显示，芬太尼是外科及创伤ICU患者发生谵妄的危险因素^【Error: Reference source not found】^；但也有研究结果与之恰好相反，芬太尼的使用可以降低谵妄的发生率^【Error: Reference source not found】^。而瑞芬太尼与谵妄之间的关系国内外尚无相关研究的报道。

目前关于以镇痛为基础治疗的研究已为临床实际应用积累了一些经验。一项关于单纯镇痛（在给予瑞芬太尼镇痛基础上必要时给予丙泊酚镇静治疗）与传统镇静联合镇痛（给予丙泊酚或咪唑安定镇静基础上必要时给予阿片类药物镇痛）治疗相比，单纯镇痛组缩短ICU滞留天数和无呼吸机天数，并使镇静镇痛评分（Sedation-Agitation Scores）明显改善【[[13]](#endnote-14)】。其他有关比较以镇痛为基础的镇静治疗与传统镇静联合镇痛治疗的多中心研究结果均显示前者可以明显缩短机械通气时间【[[14]](#endnote-15)】【[[15]](#endnote-16)】。一项最近的单中心随机对照研究，对单纯吗啡镇痛无镇静组与镇静组（给予吗啡负荷量后单纯泵入丙泊酚或咪唑安定）进行了比较，结果显示单纯镇痛组机械通气时间缩短，同时意外拔管率以及呼吸机相关性肺炎的发生率没有差异【[[16]](#endnote-17)】。可见镇痛治疗不容忽视并具有良好的应用价值和前景。

如前所述，苯二氮卓类药物是导致谵妄较为明确的诱因，但有效的镇痛治疗可以降低镇静药物用量，从而降低因镇静药物应用而导致的谵妄。因此可以提出这样的假设，在良好镇静的基础上给予苯二氮卓类药物进行镇静，这种不以改变镇静药物本身而基于用药方案的改变，可能会减少谵妄的发生。

二、研究目的：

本研究的主要研究目的之一为通过给予患者不同的镇痛镇静方案，并以脑功能保护作为研究重点，比较不同镇静镇痛方案下危重症患者谵妄的发生情况，从而寻求一种较为理想的镇静镇痛策略，降低谵妄的发生率，最大程度上实现靶器官保护。

三、材料和方法

1、研究设计：单中心随机对照研究

2、研究对象：（通过计算，为了使各组差异达到90%的有效性，需要88例患者，考虑到病例脱落，最终计划入选105名患者，每组各15名患者）

1）入选标准：

①入住ICU的术后患者；

②需要机械通气，预期机械通气时间超过24h；

③需要咪达唑仑镇静治疗；

④年龄大于18岁，小于85岁；

⑤获取知情同意。

2）排除标准：

①术前存在谵妄、酒精戒断症状、精神系统疾病及颅内病变的患者；

②术前使用过抗精神病药物或催眠药物、有酗酒病史及神经外科术后的患者；

③存在严重疾病，如：感染中毒性休克，急、慢性肾功能不全，肝功能ChildC级，神志昏迷不能配合的患者；

④妊娠或哺乳期妇女；

⑤已知对研究用药过敏或有其他禁忌症；

3随机分组：纳入患者按照随机数字表分为三组：分别为芬太尼组（F）、瑞芬太尼组（RF）和对照组（C）。

3.1芬太尼组（F）：芬太尼镇痛+咪唑安定镇静；

3.2瑞芬太尼组（RF）：瑞芬太尼镇痛+咪唑安定镇静；

3.3对照组（C）：生理盐水对照+咪唑安定镇静。

4用药方法：所有纳入试验的患者均按照试验方案给予试验药物，不能使用方案以外的镇痛镇静药物。

4.1 镇痛方法：入选患者根据不同分组，分别给予芬太尼、瑞芬太尼或生理盐水泵入。

（1）芬太尼组：1ug/kg/hr静脉泵入；

（2）瑞芬太尼组：1ug/kg/hr静脉泵入；

（3）生理盐水组：等同于试验用药组剂量进行泵入。

试验过程中每4小时对患者进行疼痛评估，分别采用BPS评分（表1）及CPOT评分（表2）系统，并记录患者BPS及CPOT评分结果。

4.2镇静方法：所有入选患者给予咪唑安定镇静

4.2.1药物配置： 抽取咪唑安定注射液用生理盐水稀释至浓度为1mg/ml。

4.2.2 用药方法：首先给予负荷剂量0.05mg/kg，继之以0.02-0.1mg/kg/h持续泵入；

试验过程中随时进行RASS评分（表3），并根据评分及时调整药物剂量，必要时追加咪唑安定（1mg/次），使RASS评分尽量维持在-1至-3分。

4.3药物使用终点：患者拔管或自用药开始后7天。

5、每日唤醒及谵妄的评估：每日上午8 时停用镇静药物定期唤醒。唤醒评估根据声音命令睁眼，眼光追随研究人员，握拳或点头示意，能够完成3项任务的患者被认为是清醒的，并评估谵妄，谵妄的判断采用用于ICU 患者的意识模糊评估法（CAM-ICU）（The Confusion Assessment Method for the ICU）标准（表4），首先对患者进行镇静评价，采用RASS评分如果RASS在-4以上(-3到+4)，开始评估CAM-ICU瞻望评分，1和2加3或4可以诊断为谵妄。一旦患者诊断为谵妄，给予右美托咪定治疗以减轻谵妄的症状。

8、数据记录

8.1 基线资料

(1)姓名、性别、年龄、体重、住院号、住ICU时间；

(2)入ICU诊断：外科、内科、创伤等；

(3)纳入试验前RASS评分、BPS评分；

(4)纳入试验最近24小时内APACHEⅡ评分、Childs-Pugh分级、血肌酐；

(5)纳入试验前镇痛药物、镇静药物和血管活性药物使用情况；

8.2 观察指标

8.2.1 主要指标：不同用药方案分组患者谵妄的发生率和谵妄持续时间。

8.2.2 次要指标：

(1) 28天死亡率；

(2) 平均机械通气时间；

(3) ICU留治天数；

(4)不同分组镇静药物的输注速度和使用总剂量；

(5)追加咪唑安定的总剂量；

(6)不同分组患者疼痛评分（BPS评分和CPOT评分）；

(9)HR、RR、BP、SpO2的变异度等。

参考文献

1. Kollef MH, Levy NT, Ahrens TS, Schaiff R, Prentice D, Sherman G. The use of continuous i.v. sedation is associated with prolongation of mechanical ventilation. Chest. 1998;114(2):541–548. [↑](#endnote-ref-2)
2. Flaherty JH, Rudolph J, Shay K, et al. Delirium is a serious and under-recognized problem: why assessment of mental status should be the sixth vital sign. J Am Geriatr Soc 2007;8(5):273–5. [↑](#endnote-ref-3)
3. Morandi A, Jackson JC. Delirium in the intensive care unit: a review. *Neurol Clin*. 2011;29(4):749–763. [↑](#endnote-ref-4)
4. Pisani MA, Kong SY, Kasl SV, Murphy TE, Araujo KL, Van Ness PH. Days of delirium are associated with 1-year mortality in an older intensive care unit population. *Am J Respir Crit Care Med*. 2009; 180(11):1092–1097. [↑](#endnote-ref-5)
5. Shehabi Y, Riker RR, Bokesch PM, Wisemandle W, Shintani A, Ely EW. Delirium duration and mortality in lightly sedated, mechanically ventilated intensive care patients. *Crit Care Med*. 2010;38(12):2311–2318. [↑](#endnote-ref-6)
6. Pandharipande P, Shintani A, Peterson J, etal. Lorazepam is an independent risk factor for transitioning to delirium in intensive care unit patients. Anesthesiology. 2006;104(1):21–26. [↑](#endnote-ref-7)
7. Pandharipande P, Cotton BA, Shintani A, etal. Prevalence and risk factors for development of delirium in surgical and trauma intensive care unit patients. J Trauma. 2008;65(1):34–41. [↑](#endnote-ref-8)
8. Agarwal V, O’Neill PJ, Cotton BA, etal. Prevalence and risk factors for development of delirium in burn intensive care unit patients. J Burn Care Res. 2010;31(5):706–715. [↑](#endnote-ref-9)
9. Kapfhammer HP, Rothenhausler HB, Krauseneck T, Stoll C, Schelling G. Posttraumatic stress disorder and health-related quality of life in long-term survivors of acute respiratory distress syndrome. *Am J Psychiatry*. 2004;161(1):45–52. [↑](#endnote-ref-10)
10. Morrison RS, Magaziner J, Gilbert M, etal. Relationship between pain and opioid analgesics on the development of delirium following hip fracture. *J Gerontol A Biol Sci Med Sci*. 2003;58(1):76–81. [↑](#endnote-ref-11)
11. Rotondi AJ, Chelluri L, Sirio C, etal. Patients’ recollections of stressful experiences while receiving prolonged mechanical ventilation in an intensive care unit. *Crit Care Med*. 2002;30(4):746–752. [↑](#endnote-ref-12)
12. Dahaba AA, Grabner T, Rehak PH, List WF, Metzler H. Remifentanil versus morphine analgesia and sedation for mechanically ventilated critically ill patients: a randomized double blind study. Anesthesiology. 2004;101(3):640–646. [↑](#endnote-ref-13)
13. Rozendaal FW, Spronk PE, Snellen FF, etal. Remifentanil-propofol analgo-sedation shortens duration of ventilation and length of ICU stay compared to a conventional regimen: a centre randomised, cross-over, open-label study in the Netherlands. *Intensive Care Med*. 2009;35(2): 291–298. [↑](#endnote-ref-14)
14. Breen D, Karabinis A, Malbrain M, etal. Decreased duration of mechanical ventilation when comparing analgesia-based sedation using remifentanil with standard hypnotic-based sedation for up to 10days in intensive care unit patients: a randomised trial [ISRCTN47583497]. *Crit Care*. 2005;9(3):R200–R210. [↑](#endnote-ref-15)
15. Karabinis A, Mandragos K, Stergiopoulos S, etal. Safety and efficacy of analgesia-based sedation with remifentanil versus standard hypnotic-based regimens in intensive care unit patients with brain injuries: a randomised, controlled trial [ISRCTN50308308]. *Crit Care*. 2004; 8(4):R268–R280. [↑](#endnote-ref-16)
16. Strom T, Martinussen T, Toft P. A protocol of no sedation for critically ill patients receiving mechanical ventilation: a randomised trial. *Lancet*. 2010;375(9713):475–480. [↑](#endnote-ref-17)
