# Supplementary figures and images for: The influence of analgesic-based sedation protocols on delirium and outcomes in critically ill patients: A randomized controlled trial
Source: PLoS One. 2017 Sep 14;12(9):e0184310. doi: 10.1371/journal.pone.0184310 (PMC5598969; doi:10.1371/journal.pone.0184310)

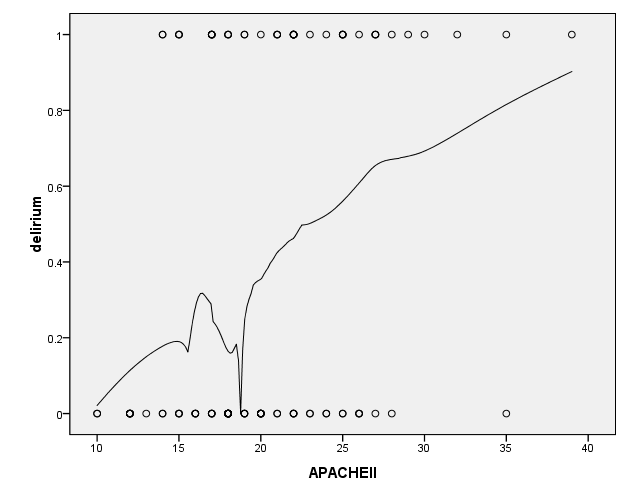

Supplement: S1 Fig — (PNG) [file pone.0184310.s005.png]

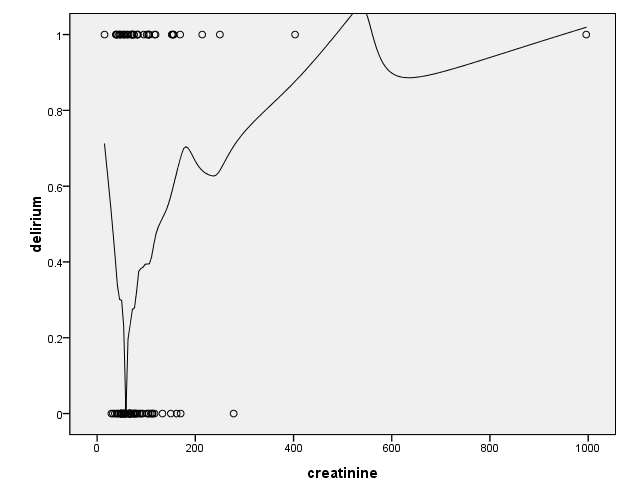

Supplement: S2 Fig — (PNG) [file pone.0184310.s006.png]

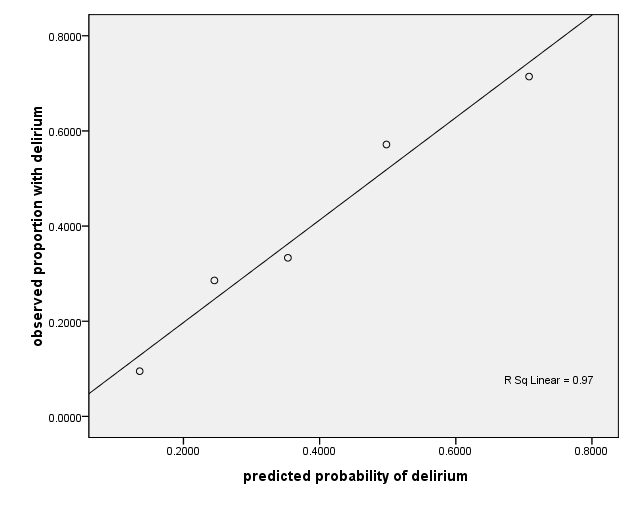

Supplement: S3 Fig — (PNG) [file pone.0184310.s007.png]
